# Lipopolysaccharide-Induced Expression of Microsomal Prostaglandin E Synthase-1 Mediates Late-Phase PGE_2_ Production in Bone Marrow Derived Macrophages

**DOI:** 10.1371/journal.pone.0050244

**Published:** 2012-11-30

**Authors:** Lei Xiao, Magdalena Ornatowska, Guiqing Zhao, Hongmei Cao, Rui Yu, Jing Deng, Yongchao Li, Qiong Zhao, Ruxana T. Sadikot, John W. Christman

**Affiliations:** 1 Department of Medicine, University of Illinois at Chicago, Chicago, Illinois, United States of America; 2 Department of Medicinal Chemistry and Pharmacognosy, University of Illinois at Chicago, Chicago, Illinois, United States of America; 3 Center for Cardiovascular Research, University of Illinois at Chicago, Chicago, Illinois, United States of America; 4 Department of Medicine, Northwestern University, Chicago, Illinois, United States of America; 5 Department of Medicine, University of Florida and Department of Veterans Affairs, Gainesville, Florida, United States of America; 6 The Jesse Brown Veterans Affairs Medical Center, Chicago, Illinois, United States of America; Beijing Institiute of Otolaryngology, China

## Abstract

Cyclooxygenase (COX)-2 expression and release of prostaglandins (PGs) by macrophages are consistent features of lipopolysaccharide (LPS)-induced macrophage inflammation. The two major PGs, PGE_2_ and PGD_2_, are synthesized by the prostanoid isomerases, PGE synthases (PGES) and PGD synthases (PGDS), respectively. Since the expression profile and the individual role of these prostanoid isomerases-mediated inflammation in macrophages has not been defined, we examined the LPS-stimulated PGs production pattern and the expression profile of their synthases in the primary cultured mouse bone marrow derived macrophages (BMDM). Our data show that LPS induced both PGE_2_ and PGD_2_ production, which was evident by ∼8 hrs and remained at a similar ratio (∼1∶1) in the early phase (≤12 hrs) of LPS treatment. However, PGE_2_ production continued increase further in the late phase (16–24 hrs); whereas the production of PGD_2_ remained at a stable level from 12 to 24 hrs post-treatment. In response to LPS-treatment, the expression of both COX-2 and inducible nitric oxide synthase (iNOS) was detected within 2 to 4 hrs; whereas the increased expression of microsomal PGES (mPGES)-1 and a myeloid cell transcription factor PU.1 did not appear until later phase (≥12 hrs). In contrast, the expression of COX-1, hematopoietic-PGDS (H-PGDS), cytosolic-PGES (c-PGES), or mPGES-2 in BMDM was not affected by LPS treatment. Selective inhibition of mPGES-1 with either siRNA or isoform-selective inhibitor CAY10526, but not mPGES-2, c-PGES or PU.1, attenuated LPS-induced burst of PGE_2_ production indicating that mPGES-1 mediates LPS-induced PGE_2_ production in BMDM. Interestingly, selective inhibition of mPGES-1 was also associated with a decrease in LPS-induced iNOS expression. In summary, our data show that mPGES-1, but not mPGES-2 or c-PGES isomerase, mediates LPS-induced late-phase burst of PGE_2_ generation, and regulates LPS-induced iNOS expression in BMDM.

## Introduction

The prostaglandins (PGs) are a group of biologically active lipid compounds that are derived enzymatically from arachidonic acid or other polyunsaturated fatty acids, and mediate a variety of important physiological and pathophysiological functions *in vivo* [1], [2]. Arachidonic acid is released from the cell membrane by phospholipase A_2_, and is converted to unstable intermediate products PGG_2_ and PGH_2_, the precursor of the series-2 prostanoids, by rate limiting COX enzymes including COX-1 and COX-2 [3], [4]. COX-1 is reported as the constitutively expressed isoform in most mammalian cells, and its role has been reported in tumorigenesis [5]. COX-2 is the inducible COX isoform, and is rarely expressed in quiescent cells. However, COX-2 is expressed in a wide range cell types (e.g., macrophages) following appropriate stimulation including growth factors, tumor promoters, hormones, and inflammatory agents [6]. COX contains two active sites: a COX site, where arachidonic acid is converted into PGG_2_; and a heme site with peroxidase activity, responsible for the reduction of PGG_2_ to PGH_2_ [3]. PGH_2_ serves as the common substrate for subsequent distal prostanoid isomerases including PGES and PGDS to generate various bioactive PGs *in vivo* [6], [7]. The production profiles of PGE_2_ and PGD_2_ in specific cells are dependent on both the stimulus and the cell type. Alteration of the production profiles of PGs *in vivo* (e.g., PGE_2_ vs. PGD_2_ ratio) is a critical determinant in the development of many diseases including cancer [8], atherosclerosis [9], arthritis [10], and pulmonary diseases [11], [12]. PGE_2_ and PGD_2_ are the two major PG isoforms involved in many inflammatory and pulmonary diseases including chronic obstructive pulmonary disease [13], bronchiectasis [14], and bronchial asthma [11]. PGD_2_ is reported to have strong proinflammatory and bronchoconstrictive action in human and animal models of asthma [15]; whereas PGE_2_ detected in bronchoalveolar lavage fluid of asthmatic patients appears to be bronchoprotective and anti-inflammatory [16]. Therefore, understanding the type, amount, source, and timing of the PGD_2_ or PGE_2_ production in the microenvironment of the diseased organs or tissues is critical for determining the precise roles of each PG isoform in the pathogenesis of many inflammatory and pulmonary diseases.

PGD_2_ is produced from PGH_2_ *in vivo* by one of the two PGDS isomerases including lipocalin-PGDS (L-PGDS) and H-PGDS [17], [18]; whereas PGE_2_ is produced from PGH_2_ by three PGES isomerases including mPGES-1, mPGES-2 and c-PGES [19]–[21]. mPGES-1 is a membrane-associated enzyme with glutathione-dependent activity, and its expression is highly inducible in response to inflammatory stimuli [22], [23]. mPGES-2 is also a membrane-associated enzyme, but doesn't require glutathione for its catalytic activity. The third PGES isomerase c-PGES is also a glutathione-dependent enzyme, and is expressed in the cytosol of a wide variety of tissues and cells [7]. H-PGDS is widely distributed in the peripheral tissues and is localized in the antigen-presenting cells, mast cells, and megakaryocytes [24]. Recently, we showed the critical role of H-PGDS isomerase in mediating LPS-induced PGD_2_ production in BMDM [25].

PU.1 is an ETS transcription factor expressed in a wide variety of hematopoietic cells including most of the myeloid cells [26]. The critical roles of PU.1 in macrophage maturation and inflammatory response to LPS had been reported by others and us [26], [27]. It also has been well documented that many inflammatory stimuli can induce iNOS expression in a variety of cells including macrophages in various inflammatory diseases [28].

We and others have previously reported that LPS-induced production of PGD_2_ and PGE_2_ in macrophages including RAW294.7 cells and BMDM can be precisely quantified by a highly sensitive and selective liquid chromatography–tandem mass spectrometry (LC–MS-MS) method [4], [29], [30]. The overall purpose of this study was to precisely characterize the production patterns and the signaling mechanisms of the inflammatory mediators PGs, and to define the complete expression profile of PGs biosynthesis-related enzymes including the involved PGES isomerases in the primary cultured BMDM, which is the most commonly used primary cell model for studying macrophage functions in the development of macrophage-related inflammatory diseases.

## Materials and Methods

### Materials

Lipopolysaccharide (LPS) and NS-398 were purchased from Sigma (St. Louis, MO). CAY10526, antibodies for COX-1, COX-2, c-PGES, mPGES-1, mPGES-2, H-PGDS were from Cayman Chemical, Inc. (Ann Arbor, MI). The PU.1 antibody was purchased from Cell Signaling (Beverly, MA). The anti-iNOS antibody was purchased from BD Biosciences (San Jose, CA). The anti-β-actin antibody was purchased from Santa Cruz Biotechnology (Santa Cruz, CA). All TaqMan gene expression assays for real-time RT-PCR studies were purchased from Life Technologies (Grand Island, NY). The ON-TARGET *plus* siRNA's for all 3 PGES isoforms and their control siRNA were purchased from Dharmacon RNAi Technologies (Thermo Scientific). The Amaxa mouse macrophage nuclefector kit was purchased from Lonza (Switzerland).

### BMDM isolation and culture

Wild-type C57BL/6 mice were obtained from Harlan (Indianapolis, IN). All procedures and protocols using mice were approved by the animal care committee (ACC) of the University of Illinois at Chicago (UIC) and the Jesse Brown Veterans Affairs Medical Center. BMDM were isolated from adult C57BL/6 mice as we previously described [32]. Briefly, after mice were euthanized, bone marrow was flushed from the femurs. The cells were washed and resuspended in DMEM medium containing 10% endotoxin-free fetal bovine serum (FBS) and 10% (vol/vol) L929 cell-conditioned medium as a biological source of macrophage colony-stimulating factor. The medium was then replenished at Day 4 in culture, and the nonadherent cells were removed. The adherent bone marrow cells were split and plated at a density of 1×10^6^/plate into P60 culture plates, and were used for the experiments after Day 7 in culture, corresponding to a mature macrophage phenotype.

### siRNA transfection

Primary cultured BMDM were transfected with 25 nM either ON-TARGET *plus* control siRNA or siRNA for PGES isoforms including mPGES-1, mPGES-2 and c-PGES using the Amaxa mouse macrophage nuclefector kit and transfection protocol (Catalog # VPA-1009, Lonza, Switzerland). After 36 hrs, BMDM were stimulated by LPS (1 µg/ml) for 16 h.

### The *in vitro* cell-free enzyme assay

The method is similar to our previous reports using recombinant mPGES-1 and COX-2 enzymes [30], [31]. Briefly, the induced COX-2 and mPGES-1 enzymes were immunoprecipitated (IP, 2 hrs at 4°C) separately from equal amount of BMDM cell lysates at either 8 hrs or 16 hrs of LPS treatment under the same experimental condition. The IP COX-2 or mPGES-1 enzyme concentration was determined using Western blotting, and equal amount of IP COX-2 or mPGES-1 enzyme from each time point was used to determine their enzyme activity of PGE_2_ production from their upstream substrates. COX-2 or mPGES-1 enzymes were incubated in 0.2 ml Tris•HCl buffer (pH 8.0 at 37°C) *in vitro* with their enzyme substrates arachidonic acid (for COX-2) or PGH_2_ (for mPGES-1), respectively. After 30 min incubation at 37°C, the reaction was stopped by adding 50 µl 2 M HCl, and the reaction end product PGE_2_ was extracted from each incubation mixture and PGE_2_ concentration was determined to compare their enzyme activities between 8 or 16 hrs. For COX-2 enzyme activity assay [31], the IP-COX-2 enzymes from BMDM were incubated with 5 µM arachidonic acid in the presence of 1 µM hematin (COX-2 co-factor), 10 µl recombinant human mPGES-1 (Cayman), and 2.5 mM GSH (mPGES-1 co-factor). For mPGES-1 enzyme activity assay [30], the IP-mPGES-1 enzymes from BMDM were incubated with 2 µM PGH_2_ in the presence of 2.5 mM GSH. PGE_2_ was then extracted from each of the above incubation mixture using 800 µL hexane/ethyl acetate (50∶50, v/v). The organic phase was removed, evaporated to dryness, and reconstituted in 100 µL methanol/water (50∶50, v/v) for analysis using LC-MS-MS (see details in the next section).

### Mass spectrometry

After the LPS treatment, the BMDM culture medium was collected and immediately stored at −80°C. The concentration of PGE_2_ and PGD_2_ in the collected culture medium was quantified by liquid chromatography in conjunction with mass spectrometry (LC-MS-MS) as we previously described [4], [30]. Briefly, HPLC separations were carried out using a Shimadzu (Columbia, MD) Prominence HPLC system with a Waters (Milford, MA) XTerra MS C_18_ (2.1 mm×50 mm, 3.5 µm) analytical column and a 5-min isocratic mobile phase consisting of acetonitrile/aqueous 0.1% formic acid (37∶63, v/v) at a flow rate of 200 µl/min. The HPLC system was interfaced to a Thermo-Finnigan (San Jose, CA) TSQuantum triple quadruple mass spectrometer that was operated using negative ion electrospray. Isomeric PGD_2_ and PGE_2_ were measured using a SRM transition of *m/z* 351 to *m/z* 271, and the SRM transition of *m/z* 355 to *m/z* 275 was selected for the internal standards d_4_-PGE_2_ and d_4_-PGD_2_.

### Western blot analysis

The method is similar to that we described previously [33]–[35]. Briefly, BMDM were lysed in lysis buffer (1% Triton X-100, 150 mM NaCl, 10 mM Tris, pH 7.4, 1 mM EDTA, 1 mM EGTA, 0.4 mM phenylmethylsulfonyl fluoride, 0.5% Nonidet P-40), and sonicated for 2 seconds to shear DNA. Soluble lysates were separated by microcentrifugation, and volume representing equal amount of proteins were boiled at 95°C for 5 min before being resolved by 10% SDS-polyacrylamide gel electrophoresis (SDS-PAGE). The proteins were transferred to PVDF membrane (Amersham Pharmacia Biotech), blocked with 5% non-fat dry milk for 1 h at room temperature, and then incubated with primary antibody at 4°C overnight. Protein was detected with horseradish peroxidase conjugated secondary antibody (Santa Cruz) and SuperSignal chemiluminescent substrate solution (Pierce). The protein loading of each sample was verified by staining the membrane with 0.1% Ponceau S solution.

### Quantitative real-time reverse transcription polymerase chain reaction (RT-PCR)

Real-time PCR was conducted using ABI Prism 7900HT Fast Real-Time PCR System (Applied Biosystems, Foster City, CA) according to the manufacturer's instructions as we previously reported [36]. The mRNA expression levels of targeted genes and an endogenous housekeeping gene encoding for β-actin as an internal control, were quantified using TaqMan gene expression assays (Life Technologies). Amplification of specific PCR product was detected using the TaqMan Fast Real-Time PCR Universal Master Mix (Life Technologies). The quantitative real-time PCR was performed in duplicate in a total reaction volume of 20 µl according to manufacturer's instruction. Blank and positive controls (calibrators) were run in parallel to determine amplification efficiency within each experiment. During the extension step, the ABI Prism 7900HT Sequence Detection System monitored PCR amplification in real time by quantitative analysis of the emitted fluorescence. Each run was completed with a melting curve analysis to confirm the specificity of amplification and lack of primer dimmer. Quantification was performed using the ΔΔCt method. The target amount of each mRNA sample was subsequently divided by the control gene amount (which was assigned a value of 1 arbitrary unit) to obtain a normalized target value.

### Confocal microscopy

BMDM were cultured in Nunc Lab-Tek II 8-well chamber slide (Fisher Scientific, Pittsburgh, PA) at 37°C and treated with or without LPS for various periods of time. BMDM were washed with 1× PBS and fixed for 20 min in 4% paraformaldehyde in PBS on ice and permeablized by 0.1% Tween 20 at room temperature (RT) for 5 min. After incubation with primary antibodies, BMDM were washed and incubated with Alexa Fluro 488-labled secondary antibodies (Life Technologies) for one hour at RT. After washing, the chamber slide was mounted with cover slips using Vectashield mounting medium (Vector Laboratories, Burlingame, CA) containing 4′, 6′ diamidino-2-phenylindole (DAPI) to stain the nucleus. Confocal microscopy images were acquired from the Carl Zeiss LSM 510 laser scanning confocal microscope equipped with a 63× water-immersion objective. Beams of 488 nm from Ag/Kr laser and 361 nm from UV laser were used for excitation. Green and blue emissions were detected through LP505 and 420 filters, respectively. The two different fluorochromes were scanned sequentially by using multi-tracking function to avoid any bleed through between the two dyes.

### Statistical analysis

All data are reported as mean ± SEM. Significance tests (student's *t*-test or ANOVA) were performed using the Prism program (GraphPAD Software). A *p* value<0.05 was considered to be significant.

## Results

### LPS stimulated expression of iNOS/COX-2/mPGES-1/PU.1 in BMDM

**Figure 1:**
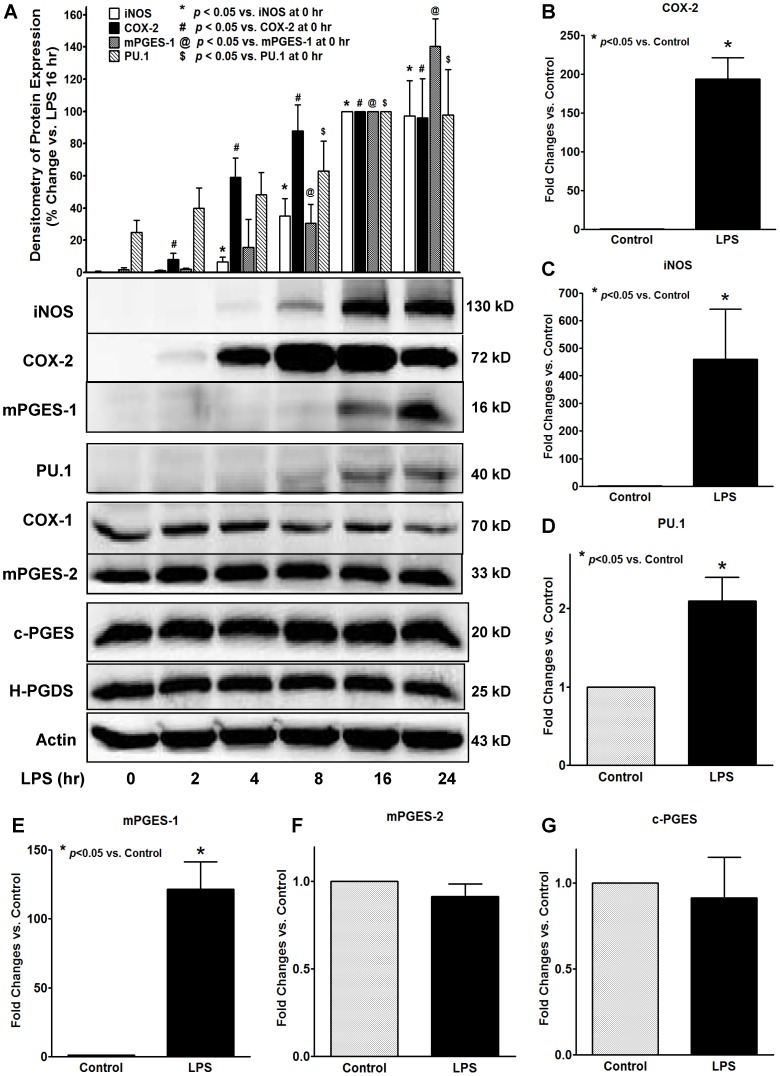
LPS induces increased expression of iNOS/COX-2/mPGES-1/PU.1 in BMDM. Primary cultured mouse BMDM was treated with or without 1 µg/ml LPS for various time points from 2 to 24 hrs. ***A***. The protein expression of targeted proteins in BMDM cell lysates was detected by Western blot assays. Representative blots and the densitometry of iNOS/COX-2/mPGES-1/PU.1 protein expression are showed from 5 independent experiments. Protein expression of COX-2 and iNOS was detected within 2 to 4 hrs post-LPS treatment; whereas increased protein expression of mPGES-1 and PU.1 was detected after 8 hrs of LPS treatment. In contrast, the protein expression of COX-1, mPGES-2, c-PGES or H-PGDS was not affected by LPS treatment. The protein loading in each experiment was normalized by β-actin. ***B–G***. The LPS-stimulated (16 hrs) mRNA expression of COX-2 (***B***), iNOS (***C***), PU.1 (***D***), mPGES-1 (***E***), mPGES-2 (***F***), or c-PGES (***G***) in BMDM was confirmed by real-time quantitative RT-PCR. The mRNA of each gene was normalized to the β-actin mRNA expression level in the same sample. The results shown are mean of at least 4 independent experiments for each gene, * *p*<0.05 vs. control.

**Figure 2:**
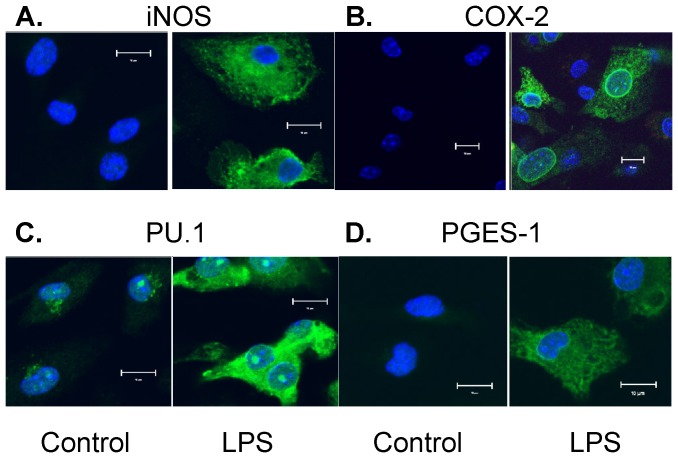
Confocol fluorescent microscopy detects the expression and intracellular distribution of iNOS/COX-2/PU.1/mPGES-1 induced by LPS treatment. Primary cultured mouse BMDM was treated with or without 1 µg/ml LPS for 16 hrs. The protein expression of iNOS (***A***), COX-2 (***B***), PU.1 (***C***), and mPGES-1 (***D***) was determined by immunostaining followed by confocol fluorescent microscopy. In each sample group, BMDM was stained with the green fluorescent-labeled antibody for the targeted protein and the blue fluorescent-labeled DAPI for nucleus; the overlay image of each targeted protein and nucleus are shown (magnification: ×200, scale bar: 10 µM). LPS stimulation significantly increased cytosolic expression of iNOS, COX-2, mPGES-1 and PU.1 in BMDM, suggesting newly synthesized protein expression in cytosol. PU.1 also showed strong nuclear staining (i.e., nuclear translocation) after LPS treatment; whereas both COX-2 and mPGES-1 showed similar enhanced perinuclear localization in LPS-treated groups. The results shown are representative images from 3 independent experiments.

We first determined the protein expression profiles of a series of inflammation-related enzymes or transcription factor in primary cultured BMDM by Western blot assays, including enzymes related to macrophage inflammation (i.e., iNOS), PG biosynthesis (i.e., COX, PGES, and PGDS isoforms), and a transcription factor for macrophage maturation (i.e., PU.1). Since our previous studies had shown that LPS concentration-dependently (0.01 to 5 µg/ml) induced the production of both PGE_2_ and PGD_2_ in BMDM with a plateau level of PGs production at about 1 µg/ml [25], we used 1 µg/ml LPS for our current studies. Among the tested proteins, LPS significantly increased the protein expression of iNOS, COX-2, mPGES-1 and PU.1 in BMDM (Figures 1A, 2A–2D). Interestingly, the time-course results showed a distinct expression pattern of the above 4 inducible proteins in BMDM. LPS-induced protein expression of iNOS (∼4 hrs) and COX-2 (∼2 hrs) occurred earlier within 4 hrs of treatment; whereas the induction of the mPGES-1 and PU.1 protein expression did not appear until 12 hrs after LPS treatment (Figure 1A). In contrast, the other PGs synthases including COX-1, mPGES-2, c-PGES and H-PGDS were constitutively expressed in BMDM, and their protein expressions were not affected by LPS treatment (Figure 1A). The mRNA expression of iNOS, COX-2, mPGES-1 and PU.1 post-LPS treatment showed similar induction patterns (Figure 1B) as that seen for their protein expression (Figure 1A). Similarly, the protein expression profile and intra-cellular distribution of the above inducible proteins were also determined by immunostaining followed by confocol microscopy (Figure 2). LPS significantly increased the cytosolic protein staining for iNOS, COX-2, mPGES-1 and PU.1 (Figure 2), suggesting these are newly synthesized proteins in the cytosol in response to LPS treatment. In addition, we also found that LPS induced enhanced staining of both mPGES-1 and COX-2 in the perinuclear area of BMDM (Figure 2B and 2D), suggesting a potential protein-protein interaction of COX-2 and mPGES-1 enzymes working collectively in generation of PGE_2_. Unlike mPGES-1, although the expressions of the other two PGES isoforms mPGES-2 and c-PGES were detected in both LPS-treated and untreated BMDM, their protein and mRNA expressions were not induced or affected by LPS stimulation (Figures 1A, 1F, 1G) and they were diffusely expressed within the cytoplasmic compartment (data not shown).

### Patterns of LPS-stimulated PGE_2_ and PGD_2_ productions in BMDM

**Figure 3:**
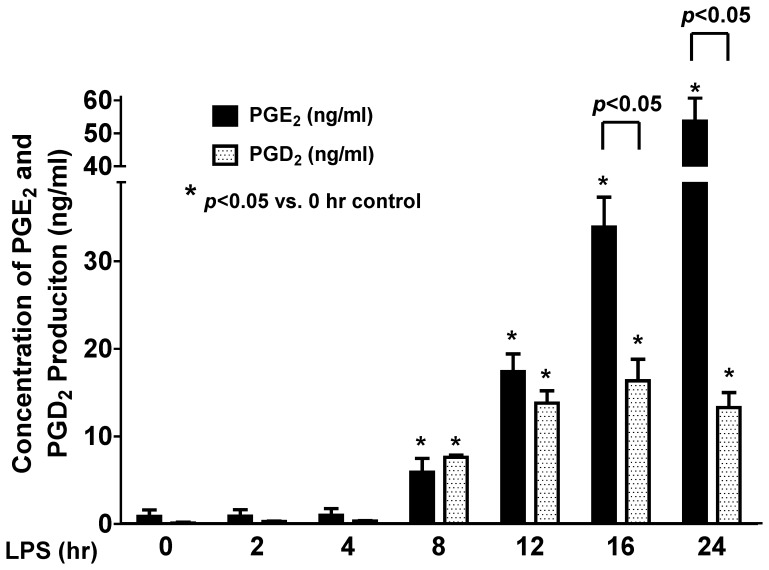
LPS stimulates PGE_2_ and PGD_2_ production in BMDM. Equal amount of BMDM was treated with or without 1 µg/ml LPS for various time points from 2 to 24 hrs. The BMDM culture medium was collected and the concentrations of PGE_2_ and PGD_2_ in the sample media were quantified using LC-MS-MS. LPS induced both PGE_2_ and PGD_2_ production at a similar lower level in the first 8 hrs of treatment. In contrast, the production of PGE_2_ in BMDM continuously increased after LPS treatment for 12 hrs; whereas the production of PGD_2_ kept at a relatively stable level after 12 hrs of LPS treatment (n = 5).

After LPS (1 µg/ml) treatment, the BMDM culture medium at each treatment time point was collected and frozen immediately at −80°C. The PGE_2_ and PGD_2_ concentration in the sample medium was quantified using LC-MS-MS method as we previously reported [4], [30]. LPS did not induce any detectable PGs production within the first 4 hrs of treatment, and the productions of both PGE_2_ and PGD_2_ were similar at 8 hrs (Figure 3). Although the production of PGE_2_ and PGD_2_ continued increase after 8 hrs, the ratio of PGE_2_ and PGD_2_ production was kept at ∼1∶1 between 8 and 12 hrs post-LPS treatment. However, the production of PGE_2_ significantly and continuously increased after 12 hrs; whereas the production of PGD_2_ stayed relatively stable from 12 to 24 hrs (Figure 3). These results indicated that although LPS-stimulated the production of both PGE_2_ and PGD_2_, their production patterns and signaling mechanisms are different in BMDM. The production of the purported pro-inflammatory PGD_2_ reached to a plateau level at about 12 hrs post-LPS treatment; whereas the production ability of the reported anti-inflammatory PGE_2_ still had not reached to its maximal level in BMDM.

### mPGES-1 selective inhibitor CAY10526 attenuated LPS-induced PGE_2_ production and iNOS expression in BMDM

**Figure 4:**
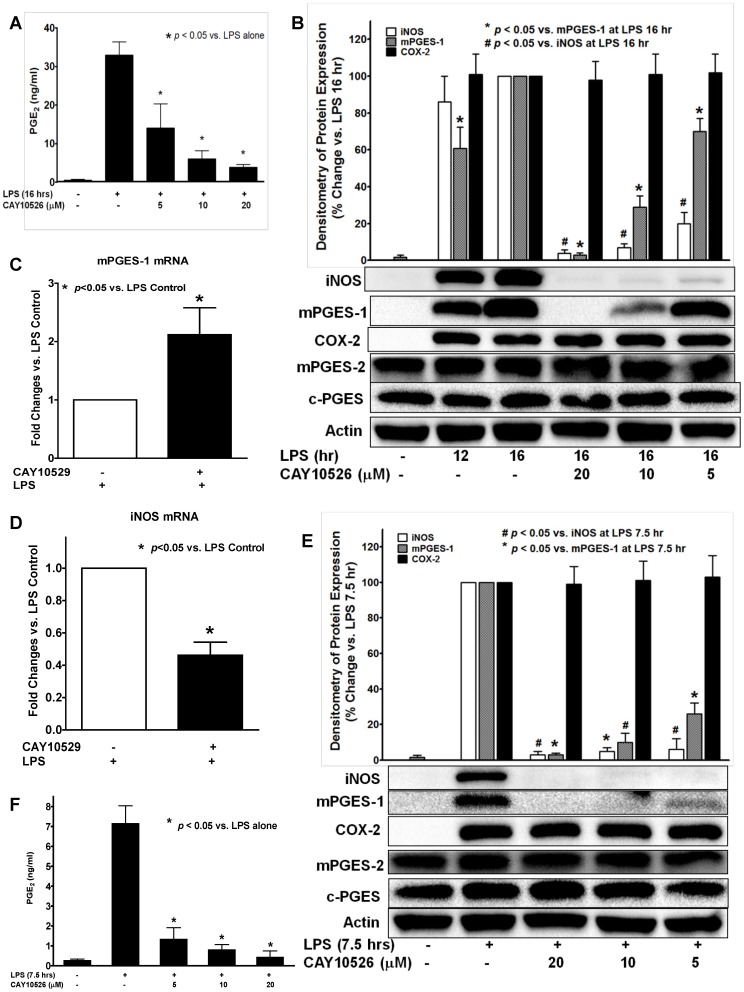
mPGES-1-selective inhibitor CAY10526 concentration-dependently attenuated LPS-induced PGE_2_ production in BMDM. BMDM was pretreated with mPGES-1-selective inhibitor CAY10526 at 5, 10, or 20 µM for 0.5 hr prior to LPS (1 µg/ml) treatment for either 16 hrs or 7.5 hrs. CAY10526 concentration-dependently inhibited the LPS-induced PGE_2_ production at 16 hrs (***A***) and 7.5 hrs (*F*) as measured by LC-MS-MS assay. Results are the mean of at least 3 independent experiments.. Western blot assays showed CAY10526 concentration-dependently attenuated the LPS-induced mPGES-1 and iNOS protein expression at either 16 hrs (***B***) or 7.5 hrs (*E*) in BMDM, but had no inhibitory effect on the protein expression of mPGES-2, c-PGES or COX-2. Representative blots and the densitometry of iNOS/mPGES-1/COX-2 protein expression were showed from at least 3 independent experiments. ***C–D.*** Real time RT-PCR results showed the efforts of CAY10526 pretreatment (10 µM, 0.5 hr prior to LPS treatment) significantly attenuated the LPS-induced (16 hrs) iNOS mRNA expression in BMDM (***D***); whereas LPS-induced (16 hrs) mPGES-1 mRNA expression in BMDM was significantly increased after CAY10526 treatment (***C***), confirming that CAY10526 selectively inhibited the translation step of mPGES-1 mRNA into proteins. Results shown were mean from 4 independent experiments.

**Figure 5:**
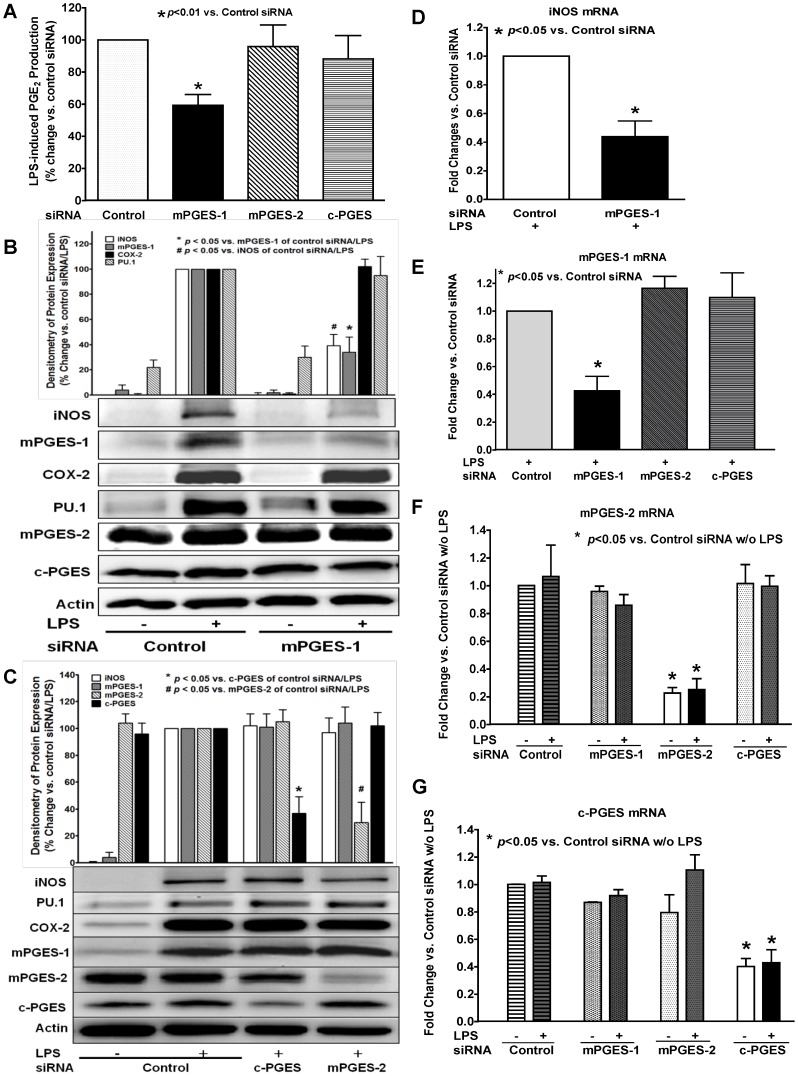
Selective siRNA inhibition of mPGES-1, but not mPGES-2 or c-PGES, attenuated LPS-induced late-phase PGE_2_ production. BMDM were transfected with siRNA's for mPGES-1, mPGES-2, c-PGES mRNA, or a control siRNA for 36 hrs, and were then treated with 1 µg/ml LPS for 16 hrs. ***A***. The PGE_2_ level in culture medium was determined by LS-MC-MC. mPGES-1 siRNA significantly attenuated LPS-induced PGE_2_ production at 16 hrs in BMDM compared to that of BMDM transfected with the control siRNA. In contrast, the siRNA for either mPGES-2 or c-PGES did not affect the LPS-induced PGE_2_ production in BMDM compared to the control siRNA group. ***B***. Western blot results showed that mPGES-1 siRNA not only selectively inhibit the protein expression of mPGES-1, but also that of iNOS. ***C***. In contrast, transfection of BMDM with siRNA's for either mPGES-2 or c-PGES selectively attenuated the expression of its targeted protein expression accordingly, but had no inhibitory effect on LPS-induced expression of iNOS, COX-2, or mPGES-1. ***D***. Real-time RT-PCR result confirmed that mPGES-1 siRNA significantly attenuated the LPS-induced mRNA expression of iNOS in BMDM at 16 hrs. ***E–G***. Real-time RT-PCR results showed that siRNA's for mPGES-1 (***E***), mPGES-2 (***F***), or c-PGES (***G***) specifically inhibited the mRNA expression of its targeted PGES isoform compared to the control siRNA group, but did not affect the mRNA expression of the other two PGES isoforms in BMDM.

Since the time course of the LPS-induced PGE_2_ production (Figure 3) highly correlated with the late-phase expression of mPGES-1 isomerase (Figure 1), we explored the potential role of the expression of mPGES-1 in the late-phase (16 hrs) burst of PGE_2_ production in BMDM. We found that the mPGES-1-selective inhibitor CAY10526 attenuated LPS-induced PGE_2_ production (Figure 4A) and protein expression of mPGES-1 in a concentration dependent manner, but did not alter the expression of other PGE_2_ synthesis-related enzymes including mPGES-2, c-PGES or COX-2 (Figure 4B), suggesting that mPGES-1 plays a critical role in the late-phase production of PGE_2_ in BMDM. Interestingly, we found that CAY10526 pretreatment significantly increased the mPGES-1 mRNA expression in BMDM as measured by real-time RT-PCR (Figure 4C). Previous reports have suggested that the inhibitory effect of CAY10526 on mPGES-1-synthesized PGE_2_ production was mediated via the selective inhibition of the mPGES-1 protein expression in RAW264 macrophages [37]. Our results confirm this finding in primary cultured BMDM and further indicate that this inhibitory effect of CAY10526 on mPGES-1 does not occur at its mRNA transcription level as these CAY10526-treated BMDM still could continue transcribe mPGES-1 mRNA without any inhibition in response to the LPS stimulation (Figure 4C). Instead, the inhibitory effect of CAY10526 possibly occurs at its mRNA translation step (leading to decreased expression of newly-synthesized mPGES-1 protein), or at the post-translational level (i.e., increasing mPGES-1 protein degradation). The increase of LPS-induced mPGES-1 mRNA transcription in the CAY10526-treated group (Figure 4C) suggested a novel positive-feedback mechanism in BMDM to overcome the loss of mPGES-1 enzyme activity and its protein expression (i.e., decreased translation of mPGES-1 mRNA into protein, or increased mPGES-1 protein degradation) due to the selective inhibition of CAY10526 on mPGES-1. Taken together, these data showed that the CAY10526 inhibited late-phase PGE_2_ production in BMDM was mediated via the selective inhibition of LPS-induced expression of mPGES-1, but not PGES-2, c-PGES, or COX-2. Surprisingly, we observed that CAY10526 pretreatment also attenuated LPS-induced expression of iNOS both at protein (Figure 4B) and mRNA (Figure 4D) levels in BMDM, but had no inhibitory effect on the other PGE_2_ synthesis-related enzymes including COX-2, mPGES-2 or c-PGES (Figure 4B). This result suggests that the mPGES-1 expression or mPGES-1-mediated PGE_2_ production contributes to the further induction of late-phase iNOS expression in BMDM. This finding was subsequently confirmed by our data using mPGES-1 siRNA (Figure 5B). In comparison, we also tested the effect of mPGES-1 inhibition on early phase (<8 hrs) LPS-induced iNOS expression in BMDM. In order to ensure the sufficient and detectable mPGES-1 expression and PGE_2_ production by LPS treatment, we chose 7.5 hrs as the early phase time point. Like in the late phase of LPS treatment (16 hrs), the mPGES-1-selective inhibitor CAY10526 also concentration-dependently inhibited both mPGES-1 and iNOS expression (Figure 4E), as well as the LPS-induced PGE_2_ production in BMDM (Figure 4F), suggesting the critical role of mPGES-1 in both earlier and late phase LPS-induced PGE_2_ production in BMDM.

### Selective siRNA inhibition of mPGES-1, but not mPGES-2 or c-PGES isomerase, prevented LPS-induced late-phase PGE_2_ production

To more precisely show a role for the various PGES isomerases in LPS-induced late-phase PGE_2_ production in BMDM, we used siRNA transfection method to test the effect of each of the three PGES isomerases on LPS-induced PGE_2_ production. siRNA's for mPGES-1, mPGES-2, c-PGES mRNA, and a control siRNA were used to transfect the BMDM prior to LPS treatment. mPGES-1 siRNA selectively inhibited LPS-induced expression of both mPGES-1 (Figures 5B & 5E) and iNOS (Figures 5B & 5D), and significantly attenuated the late-phase PGE_2_ production (Figure 5A). In contrast, siRNA inhibition of either mPGES-2 (Figures 5C & 5F) or c-PGES (Figures 5C & 5G) isomerase did not affect the PGE_2_ production compared to that of the BMDM transfected with the control siRNA (Figure 5A). These data indicated that mPGES-1 isomerase, but not mPGES-2 or c-PGES, mediated LPS-induced late-phase PGE_2_ production in BMDM. Furthermore, these siRNA results also confirmed our above finding with CAY10526 that mPGES-1 regulates LPS-induced iNOS expression in BMDM, as siRNA for mPGES-1, but not for mPGES-2 or c-PGES, significantly attenuated LPS-induced iNOS expression (Figures 5B & 5C). This finding is consistent with our above observation using the mPGES-1 selective inhibitor CAY10526 in BMDM.

### Selective siRNA inhibition of PU.1 in BMDM did not affect LPS-induced mPGES-1 or late-phase PGE_2_ production

**Figure 6:**
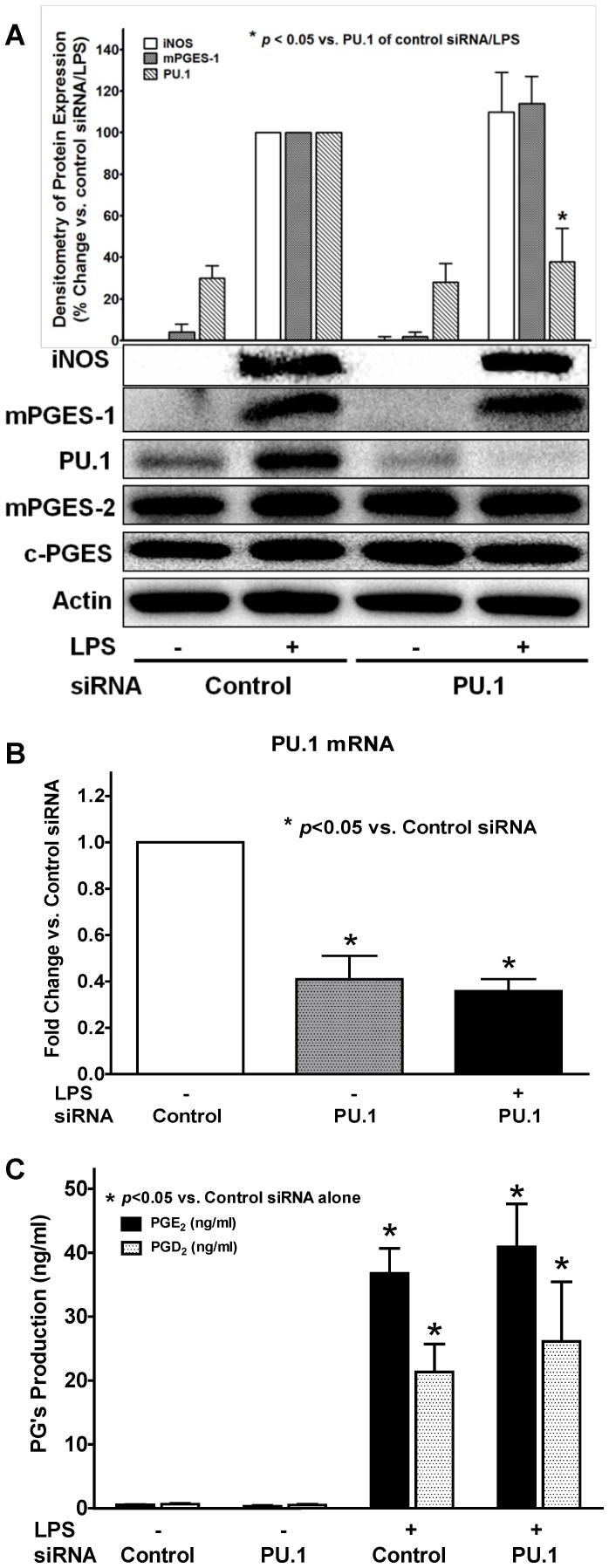
Selective inhibition of PU.1 didn't affect LPS-induced mPGES-1 expression or late-phase PGE_2_ production in BMDM. BMDM was transfected with either PU.1 siRNA or a control siRNA for 36 hrs, and were then treated with or without 1 µg/ml LPS for 16 hrs. The concentration of PGE_2_ and PGD_2_ in culture medium was then determined by LS-MC-MC. The protein and mRNA expression in BMDM was determined by Western blot assay or real-time RT-PCR, respectively. ***A***. PU.1 siRNA significantly prevented LPS-induced protein expression of PU.1 in BMDM, but had no inhibitory effect on the protein expression of iNOS, mPGES-1, mPGES-2, or c-PGES (representative blots and the densitometry of iNOS/mPGES-1/PU.1 protein expression were showed from 3 independent experiments). ***B***. Real-time RT-PCR result confirmed that PU.1 siRNA significantly attenuated the PU.1 mRNA expression with or without LPS (16 hrs) treatment in BMDM. ***C***. LPS-induced (16 hrs) PGE_2_ and PGD_2_ production in BMDM was not affected by PU.1 siRNA (n = 3).

We and others had previously reported the potential important roles of the ETS family transcriptional factor PU.1 in macrophage maturation and inflammatory response to LPS stimulation [26], [27]. In the current studies, we found LPS significantly induced PU.1 protein expression at late phase (∼16 hrs) similar to that seen in mPGES-1 expression. Thus, we explored the potential link between PU.1 expression and late-phase PGE_2_ production in response to LPS treatment using siRNA for PU.1 mRNA. We found that although PU.1 siRNA transfection significantly prevented LPS-induced expression of PU.1 at both protein (Figure 6A) and mRNA (Figure 6B) levels, it showed no inhibitory effect on either PGE_2_ production (Figure 6C) or the expression of any of the three PGES isomerases (Figure 6A). Conversely, we found that siRNA selective inhibition of any of the above three PGES isomerases also did not affect the LPS-induced PU.1 expression in BMDM (Figure 5B–5C). Therefore, our data suggested that although the LPS-induced PU.1 induction in BMDM appeared with a similar time-course (late-phase expression) to those of mPGES-1 expression (Figure 1) and PGE_2_ production burst, PU.1 expression may be parallel to or independent of the mPGES-1 expression signaling pathway, and thus may not directly contribute to the late-phase PGE_2_ production induced by LPS.

### Selective inhibition of COX-2 completely inhibited PGs production, but did not affect LPS-induced mPGES-1 expression

**Figure 7:**
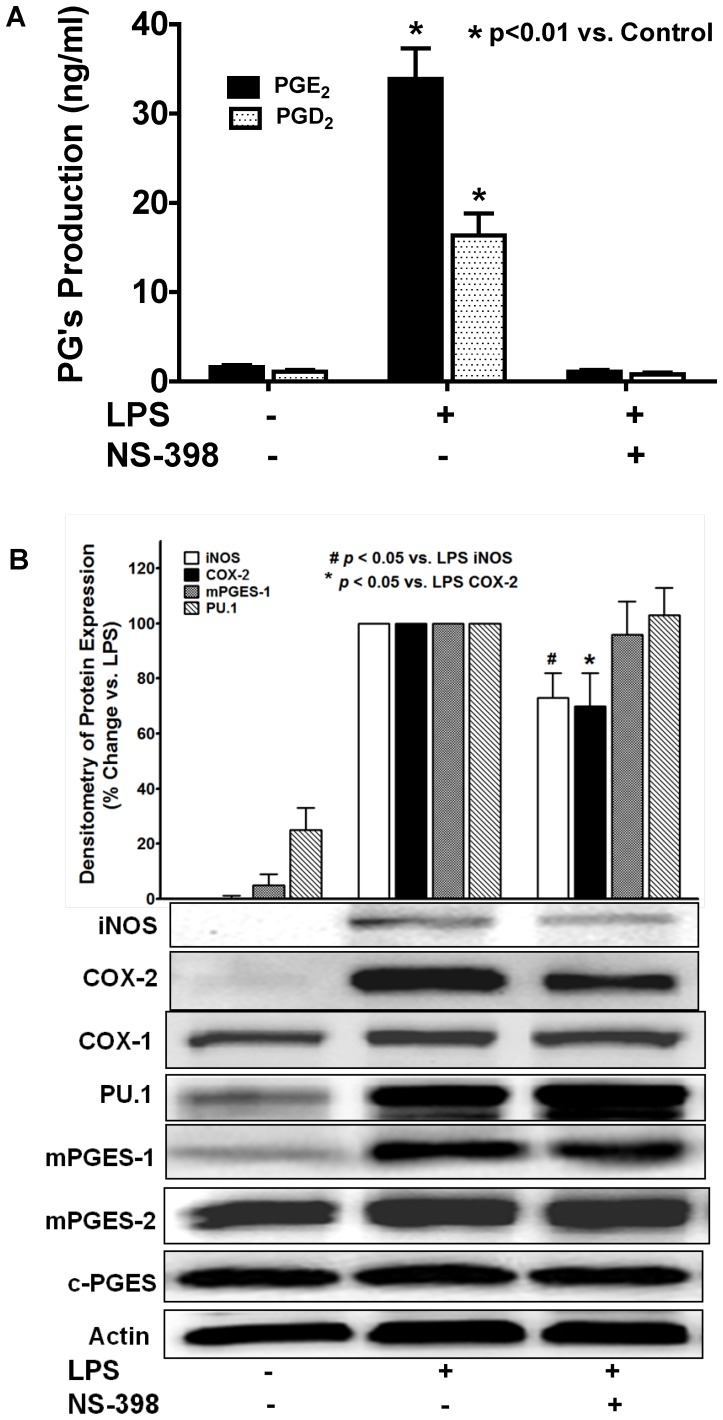
Selective inhibition of COX-2 abolished LPS-induced PGs production, but didn't affect LPS-induced mPGES-1 expression. BMDM were pretreated with COX-2-selective inhibitor NS-398 (20 µM, 0.5 hr) prior to the treatment of 1 µg/ml LPS for 16 hrs, the PGE_2_ and PGD_2_ levels in culture medium were measured by LS-MC-MC. ***A***. NS-398 completely prevented LPS-induced both PGE_2_ and PGD_2_ production (n = 3). ***B***. NS-398 also partially inhibited LPS-induced protein expression of COX-2 and iNOS, but had no inhibitory effect on the expression of COX-1/PU.1/mPGES-1/mPGES-2/c-PGES by Western blot assay. The representative blots and the densitometry of iNOS/COX-2/mPGES-1/PU.1 protein expression were from 3 independent experiments.

It has been well documented in literature that LPS-induced PGs production in macrophages is mediated via selective expression and activation of the upstream PGs synthase COX-2 [6]. In order to test the potential role of COX-2 in LPS-induced mPGES-1 expression and late-phase PGE_2_ production, BMDM were pretreated with a COX-2-selective inhibitor NS-398 prior to LPS treatment. NS-398 pretreatment completely prevented LPS-induced both PGD_2_ and PGE_2_ production (Figure 7A) in BMDM via inhibition of COX-2 enzyme activity as previously reported [38], [39] and also partial inhibition of its protein expression (Figure 7B), but showed no inhibitory effect on LPS-induced expressions of the three PGES isomerases or COX-1 (Figure 7B) in BMDM. These data suggested that LPS-induced mPGES-1 expression is not directly triggered by or dependent on the expression and activation of its immediate upstream PG synthesis enzyme COX-2. In addition, we found that NS-398 pretreatment not only prevented LPS-induced PGE_2_ production but also attenuated the iNOS expression in BMDM, which was consistent with our findings that inhibition of late-phase PGE_2_ production using mPGES-1 siRNA or CAY10526 could attenuate LPS-induced iNOS expression in BMDM.

### The enzyme activities of expressed COX-2 and mPGES-1 between early and late phase of LPS treatment were not significantly different

**Figure 8:**
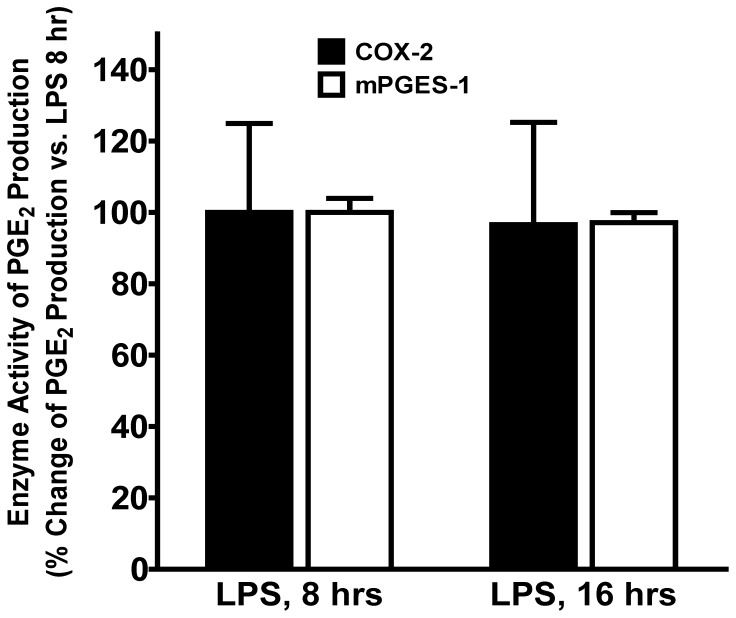
The enzyme activities of expressed COX-2 and mPGES-1 enzymes between early and late phase of LPS treatment were not different. The induced COX-2 and mPGES-1 enzymes were immunoprecipitated (IP, 2 hrs at 4°C) separately from equal amount of BMDM lysates at either 8 or 16 hrs of LPS treatment under the same experimental condition. The IP COX-2 or mPGES-1 enzyme concentrations were determined, and equal amount of IP COX-2 or mPGES-1 enzymes from each time point was used to determine their enzyme activity of PGE_2_ production from their substrates (either arachidonic acid or PGH_2_) *in vitro*. No significant difference in either COX-2 or mPGES-1 enzyme activity of PGE_2_ production was detected between 8 and 16 hrs of LPS treatment (n = 3).

Although our results clearly demonstrated the significant difference of mPGES-1 protein expression in BMDM between early and late phase of LPS treatment (Figure 1), which also correlated with the changes of PGE_2_ production (Figure 3) at different time points, it is also possible that the enzyme activities of mPGES-1 or COX-2 changed in response to LPS at different time points. In order to explore this possibility, we compared the COX-2 and mPGES-1enzyme activities between 8 and 16 hrs of LPS treatment using equal amount of IP COX-2 or mPGES-1 enzymes. The enzyme activities were determined by *in vitro* enzyme assay measuring enzyme-mediated PGE_2_ production. Our results showed (Figure 8) that there is no significant difference in either COX-2 or mPGES-1 enzyme activity of PGE_2_ production *in vitro* between 8 and 16 hrs of LPS treatment, suggesting the difference of PGE_2_ production between 8 and 16 hrs of LPS treatment was not due to the changes of COX-2 or mPGES-1 enzyme activities, but more likely because of the difference in enzyme expression levels (i.e. mPGES-1) and/or other upstream factors such as substrate availability (e.g., arachidonic acid released by PLA_2_) at early or late phase of LPS treatment.

## Discussion

PGE_2_ and PGD_2_ are the two major PGs in the human body with distinct biological functions in different tissues or cells *in vivo*. Macrophages are a major producing source of PGs and play an important role in the development of in many pulmonary and inflammatory diseases *in vivo*. BMDM are the most commonly used primary cell model to study the functions of macrophages in disease states [27], [32], [40]. However, the complete expression profile of PGs synthesis-related enzymes and the precise time courses and ratio of PGE_2_ and PGD_2_ production in response to LPS stimulation has not been previously characterized in BMDM. In the current study, we investigated the expression profile of PGs synthases and other inflammation-related proteins in BMDM; we also studied the LPS-induced PGs production patterns and the potential signaling mechanisms of the PGE_2_ production in BMDM.

We found that the LPS stimulation significantly increased the expression of inducible COX-2 and iNOS proteins similar to what we and others showed in macrophage cell line RAW294.7 [4]. The expression of these two proteins occurs relatively earlier at about 2 to 4 hrs post-LPS treatment, suggesting the direct cellular responses of synthesis of these two proteins in response to LPS stimulation. In contrast, unlike in RAW294.6 macrophages [4], the expression of prostanoid isomerase mPGES-1 and a transcription factor PU.1 in BMDM showed a distinct time course and only appeared to be inducible at a much later time (8–16 hrs) after LPS stimulation. The LPS-induced expression of the above four inducible proteins in BMDM at both protein and mRNA levels were confirmed by confocol microscopy and real-time RT-PCR, respectively. This is an interesting finding suggesting that mPGES-1 (late-phase expression) may have distinct activation mechanisms from COX-2 (early-phase expression) in response to LPS stimulation. In addition, we found that the increase in expression of mPGES-1 directly correlated with the significantly increased production of PGE_2_ at late phase post-LPS treatment in BMDM. Inhibition of mPGES-1 expression or its function by either mPGES-1-specific siRNA or mPGES-1 selective inhibitor CAY10526 prevented the LPS-induced production of PGE_2_, indicating a causal relationship between mPGES-1 expression and the burst of PGE_2_ production. In contrast, inhibition of the other two PGES isoforms including mPGES-2 and c-PGES did not affect the LPS-induced late-phase PGE_2_ production. These results clearly show a role for mPGES-1 in mediating the LPS-induced late-phase PGE_2_ production in BMDM.

Although mPGES-1 has been reported as an inducible isomerase previously [22], [23], we also found that it is constitutively expressed in RAW264.7 macrophages and not inducible by LPS treatment [4]. Therefore, our finding showed that the expression pattern and response of mPGES-1 enzyme in primary cultured BMDM are different from those in the commonly used macrophage cell line like RAW 264.7, which may explain the relatively lower production level of PGE_2_ vs. PGD_2_ production in our previous report using RAW264.7 cells [4], [36]. Our results are consistent with previous reports that the c-PGES and mPGES-2 are mainly constitutively expressed enzymes. c-PGES was reported to use PGH_2_ produced by COX-1 to generate PGE_2_ [21]; whereas mPGES-1 uses COX-2-dervived PGH_2_ as its preferred substrate [19]. mPGES-2 can use PGH_2_ generated from both sources [20]. In our studies, the mPGES-1 showed an enhanced perinuclear expression post-LPS treatment in addition to the elevated cytosolic expression using confocol microscopy, which is consistent with the COX-2 expression pattern in BMDM (Figure 2B and 2D), suggesting that the expression of these two sequential PG synthases are physically in close proximity to each other after LPS treatment. Since we found that the COX-2 protein is already highly expressed at about 4 hrs post-LPS treatment, thus BMDM should be able to generate PGH_2_ that accordingly serve as substrate for the available cellular PGES isomerases (i.e., mPGES-2 and c-PGES) at the time to produce PGE_2_ in BMDM. However, although both mPGES-2 and c-PGES isoforms are consistently present in the cytosol of BMDM, we did not detect any significant PGs production until 8 hrs post-LPS treatment. The results show that the expression of COX-2 alone with the presence of mPGES-2 and c-PGES isomerases may not be sufficient in biosynthesis of PGE_2_ in BMDM. Among the three PGES isomerases, mPGES-1 is reported to be the most efficient enzyme in catalyzing PGH_2_ to PGE_2_ (310 mM^−1^ s^−1^) [41] vs. mPGES-2 (65 mM^−1^ s^−1^) [42] or c-PGES (∼57 mM^−1^ s^−1^) [21], which is consistent with our finding of the significant increase of PGE_2_ production at late phase as soon as the mPGES-1 starts to appear. However, our results don't completely exclude a possible minor role for mPGES-2 or c-PGES in mediating the basal or low level of PGE_2_ production at the earlier phase (≤12 hrs) of LPS treatment in BMDM. In fact, since COX-2 expression already appears at 2∼4 hrs post-LPS treatment but the mPGES-1 expression was not detectable until about 8 hrs, it is likely that either mPGES-2 or c-PGES may contribute to the earlier phase basal or low level of PGE_2_ production in BMDM, as they could use the PGH_2_ generated by either the constitutively expressed COX-1 or the early expressed COX-2 enzymes to synthesize PGE_2_. Moreover, we recently reported that a portion of the COX-generated PGH_2_ (∼21%) can spontaneously covert to PGE_2_ or PGD_2_ in cell-free enzymatic assays *in vitro* without the presence of any PGDS or PGES enzymes in the reaction system [30]. We confirmed that neither COX-1 nor COX-2 has the PGES or PGDS isomerase activity *in vitro*, and thus this portion of spontaneous conversion of PGH_2_ to PGE_2_ or PGD_2_ in cell-free system is independent of COX-1 or COX-2 enzyme activity *in vitro*. It is possible that in addition to the potential roles of the mPGES-2 or c-PGES in LPS-induced early phase of PGE_2_ production, the COX-2 synthesized PGH_2_ might spontaneously convert to PGE_2_ in BMDM prior to the LPS-induced expression of mPGES-1. Nevertheless, our current studies focus on the late-phase burst of PGE_2_ production in BMDM, which generates incomparable large amount of the reported anti-inflammatory PGE_2_ and thus may potentially have more impact on its downstream signaling pathways or cellular functions in macrophages than that generated from the early phase.

Since COX-2 is the major rate-limiting upstream enzyme in PGE_2_ synthesis in response to LPS treatment in macrophages [6], we thus investigated the potential role of COX-2 in LPS-induced mPGES-1 expression and late-phase PGE_2_ production, we found that inhibition of COX-2 enzyme by its selective inhibitor NS-398 completely prevented the LPS-induced both PGE_2_ and PGD_2_ production, but did not have any effects on the LPS-induced mPGES-1 expression, suggesting that the expression of mPGES-1 is independent of COX-2 expression and the generation of PGs. Therefore, the signaling pathway of LPS-induced mPGES-1 expression in BMDM is not necessarily downstream from the COX-2 expression, but rather parallel to the signaling pathway leading to COX-2 expression, although both these two enzymes work coordinately in biosynthesis of PGE_2_ in response to LPS stimulation. In addition, our *in vitro* enzyme activity assays showed that the enzyme activities of the induced COX-2 and mPGES-1 enzymes were not different between early and late phase of LPS treatment in BMDM, suggesting the significant increase of PGE_2_ in late phase is most likely due to the LPS-induced increase of mPGES-1 protein expression in BMDM, but not increase of enzyme activities.

The role of PU.1 in macrophage maturation and LPS-stimulated inflammatory response has been previously reported by others and us [26], [27]. In the current studies, we found similar induction time-courses of PU.1 and mPGES-1 protein expression in response to LPS treatment (i.e., late-phase expression); we thus studied the potential relationship between PU.1 expression and late-phase PGE_2_ production and the potential regulatory role of PU.1 in mPGES-1 expression using siRNA method. We found that selective inhibition of LPS-induced PU.1 expression had no inhibitory effect on either PGE_2_ production or the protein expression of any of the three PGES isomerases. Conversely, selective inhibition of PGES isomerases by their siRNA's also did not affect the LPS-induced PU.1 expression in BMDM. Therefore, our results suggested that although LPS stimulated PU.1 expression in BMDM with a similar time-course to those of mPGES-1 expression and the burst of PGE_2_ production, PU.1 expression is required for neither mPGES-1 expression nor PGE_2_ production, and thus may not directly contribute to the LPS-induced late-phase mPGES-1 expression and PGE_2_ production.

Another interesting and potentially important finding of our studies is the effect of mPGES-1 expression on the LPS-induced iNOS expression in BMDM. Our data clearly showed that inhibition of mPGES-1 expression using either mPGES-1 siRNA or its selective inhibitor CAY10526 not only attenuated LPS-induced PGE_2_ production, but also prevented the iNOS expression at both protein and mRNA levels. Conversely, the expression of iNOS is strongly enhanced when the mPGES-1 expression starts to appear (Figure 1, 4B, 4E). These results were also confirmed by selective inhibition of COX-2 using NS-398, which inhibited both PGs production and the LPS-induced iNOS expression. To our knowledge, this is the first report showing the potential regulatory role of mPGES-1 in the expression of iNOS in macrophages. The expression of iNOS in phagocytes is considered as a hallmark for inflammatory response after pathogen exposure, and many inflammatory stimuli could lead to the expression of iNOS in macrophages [28]. PGE_2_ is a potent immunomodulator in inflammation, its actions in phagocytes could lead to either immunosuppressive conditions possibly via the activation of its EP2 and EP4 receptors that results in the production of cAMP [43], [44], or immunostimulatory effects via the activation of its EP3 receptor that results in decreased production of cAMP [45], [46]. We found that the burst of PGE_2_ production in BMDM is mediated via mPGES-1, and the inhibition of either mPGES-1 or COX-2 prevented PGE_2_ production and iNOS expression in BMDM. These data suggested that the PGE_2_ generated from mPGES-1 may contribute to and mediate the LPS-induced iNOS expression in BMDM. However, the precise signaling mechanism how mPGES-1 expression regulates LPS-induced iNOS expression still awaits further investigation.

In summary, our studies first determined the precise expression profile of PGs synthesis-related enzymes including the prostanoid isomerases and COX, and the production patterns of PGE_2_ and PGD_2_ in BMDM. LPS induces expression of iNOS, COX-2, mPGES-1 and PU.1 in BMDM with distinct expression time courses: The protein expression of COX-2 and iNOS appears within early phase (2–4 hrs) after LPS treatment; whereas LPS-induced mPGES-1 and PU.1 expression appears at a much later phase (after 8 hrs). LPS stimulates both PGD_2_ and PGE_2_ production in BMDM at a similar level between 8–12 hrs post-LPS treatment, but triggers significant burst of PGE_2_ production at later time (16–24 hrs). This late-phase burst of PGE_2_ production in BMDM is mediated via LPS-induced mPGES-1 expression, but not mPGES-2 or c-PGES isomerase, nor changes of enzyme activities of mPGES-1 or COX-2. Although COX-2 expression directly regulates LPS-induced production of PGE_2_ and PGD_2_, LPS-induced mPGES-1 expression in BMDM is independent of COX-2 or PU.1 expression. In addition, mPGES-1 expression directly regulates the LPS-induced iNOS expression likely via its production of PGE_2_ as its downstream mediator, because inhibition of mPGES-1 expression or PGE_2_ production prevented LPS-induced iNOS expression in BMDM. Our studies showed the unique late expression pattern of mPGES-1 in primary cultured macrophages for the first time, which mediates the burst of late-phase PGE_2_ production and regulates the iNOS expression. This late-phase burst of PGE_2_ production shifts the balance of the production of PGD_2_ and PGE_2_ in BMDM, and alters the ratio of the purported proinflammatory PGD_2_ and the anti-inflammatory PGE_2_ in macrophages, which may indicate an important *in vivo* functional adjustment in host defense in order to better manage the cellular response to bacterial infection.
